# Toward a holographic brain paradigm: a lipid-centric model of brain functioning

**DOI:** 10.3389/fnins.2023.1302519

**Published:** 2023-12-14

**Authors:** Marco Cavaglià, Marco A. Deriu, Jack A. Tuszynski

**Affiliations:** ^1^DIMEAS, Politecnico di Torino, Turin, Italy; ^2^Department of Data Science and Engineering, The Silesian University of Technology, Gliwice, Poland; ^3^Department of Physics, University of Alberta, Edmonton, AB, Canada

**Keywords:** lipid membrane, electromagnetic field, holography, action potential, consciousness

## Abstract

Due to the stimulation of neuronal membrane dipoles by action potentials, under suitable conditions coherent dipole oscillations can be formed. We argue that these dipole oscillations satisfy the weak Bose-Einstein condensate criteria of the Froehlich model of biological coherence. They can subsequently generate electromagnetic fields (EMFs) propagating in the inter-neuronal space. When neighboring neurons fire synchronously, EMFs can create interference patterns and hence form holographic images containing analog information about the sensory inputs that trigger neuronal activity. The mirror pattern projected by EMFs inside the neuron can encode information in the neuronal cytoskeleton. We outline an experimental verification of our hypothesis and its consequences for anesthesia, neurodegenerative diseases, and psychiatric states.

## Introduction

Neuroscience advanced by discovering electrical properties of the brain and its constituent signaling cells, neurons. The pioneer of neuro-electrophysiology, Galvani, discovered “animal electricity,” followed by Tesla who invented electrotherapeutic instruments. Since then, definitive evidence has been amassed about brain’s electrical activity (Renga, 2020) showing that human physiology correlates with synchronization of the neuronal electrical activity in specific brain regions (Afrasiabi et al., 2021). The brain creates physical 3D reality perception from the sensory inputs activating receptors, which subsequently generate electrical activity of billions of interconnected neurons. How physical reality is represented by the brain as a coherent and internally-consistent whole is the main aim of the theory we propose. Every animal with a nervous system creates its own physical reality, that we believe is based upon a common molecular mechanism not yet investigated in neuroscience. Addressing this issue is the first step toward understanding the emergence of qualia. Our theory focuses on the lipid composition of neuronal membrane and hypothesizes that phospholipid head groups are the main players in the electromagnetic mechanism behind 3D reality perception. As indirect evidence, weak electric and magnetic fields are known to impact brain activity and are used to treat psychiatric conditions (Chail et al., 2018; Di Iorio et al., 2022). MacIver shifted the focus from external EMF and their impact on the brain to the internally generated EMF energy cloud that could strengthen ephaptic connections to neural circuits, enhancing the interaction between various forms of energy within the brain (Mac Iver, 2022).

The molecular-level mechanism of brain functioning is based on EMF generation by excitable neuronal membranes (Baluška et al., 2021).

The neuronal action potential (AP) is generated in the axon hillock and propagates along the axonal membrane toward its terminals. Our knowledge about APs is based on the Nobel prize-winning Hodgkin-Huxley (HH) model of the non-linear propagation of voltage pulses in the giant squid axon. Electrophysiology of excitable membranes focuses on the electrical conduction properties of ion channels. Notably, the neuronal membranes of dendrites, soma, and axons exhibit distinct electrical properties. They comprise numerous types of amphipathic phospholipids whose composition, distribution and electrical permittivity determine the parameters of AP propagation such as amplitude and speed. Importantly, the HH model is one-dimensional, which ignores axon’s cylindrical geometry. This is a non-trivial aspect for nonlinear differential equations as it requires a ring-like AP wave (Singh et al., 2021).

**Figure 1:**
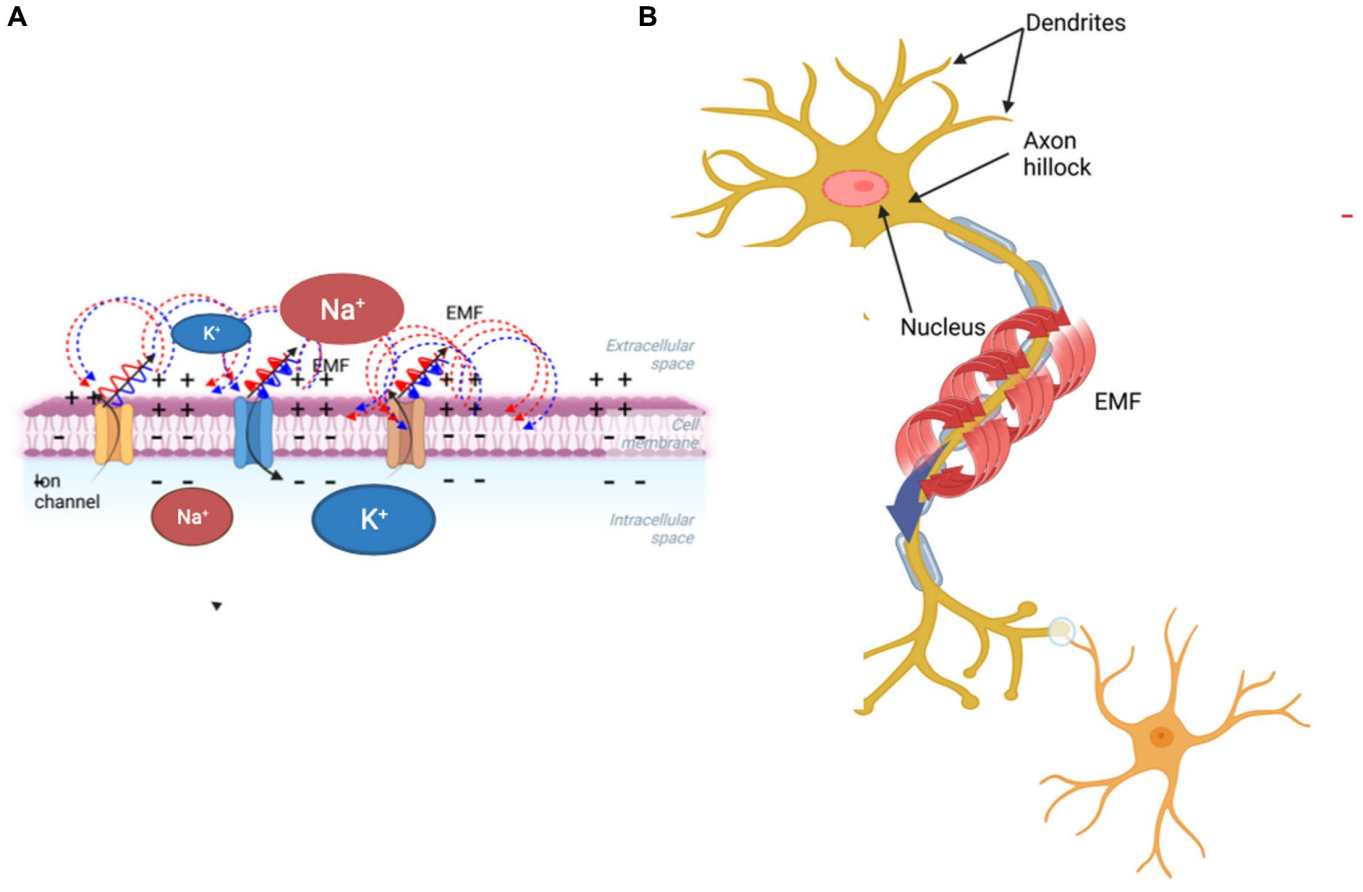
Schematic diagram showing how a propagating action potential involving ion transfer across the membrane **(A)** generates a magnetic field surrounding the axon **(B)**.

Our model describes electromagnetic (EM) energy transduction involving the external and internal lipid layers of the neuronal membrane and a subsequent integration of electrical signals resulting in physiological responses. We propose a testable physical mechanism whereby the membrane’s phospholipid head groups generate dipole oscillation, which can lead to EM effects. Their dipoles interact with their neighbors, and with the electrical potential gradients of propagating APs (Figure 1). Importantly, recent experiments determined that the membrane’s electrical and mechanical degrees of freedom are nonlinearly coupled, leading to soliton formation (Lautrup et al., 2011). Solitons are nonlinear localized waves that propagate with no loss of amplitude or velocity.

Under suitable conditions, these dipolar oscillations become synchronized and generate EMFs (Sun and Sun, 2022) in the outer and inner membrane, which might cause physiological effects at the cellular and supra-cellular levels. EMFs generated by two or more neurons may lead to constructive or destructive interference patterns containing information provided by sensory inputs. It is plausible that the brain’s electrical activity depends on the phospholipid composition, electrical capacitance, and mechanical properties of the neuronal membrane. Our model extends the HH theory by stressing the role of the phospholipid membrane in the information propagation through the complex neuronal network. Neuronal membrane composition is particularly rich with polyunsaturated docosahexaenoic acid (DHA) whose main dietary source is linoleic acid (Janssen and Kiliaan, 2014). This is pivotal to neurotransmitter exocytosis and indirectly to signal propagation and EMF generation. Molecular-level characterization of neuronal membrane phospholipids, which has been empirically implicated in psychopathology, could shed light on both the normal brain function and the etiology of neurological disorders (Sun and Sun, 2022).

## Electromagnetic, thermodynamic and mechanical properties of neurons

Electrical signals received, transmitted and processed by the brain are similar to the Morse code and utilized by the human brain to coordinate behavior, sensation, thoughts, emotions, and neurovegetative functions (Bonnstetter and Collura, 2021). Diverse spatio-temporal techniques are used to measure the human brain’s neuronal electrical activity (Nir et al., 2007) as correlates of these functions. However, a quantitative relationship between the electrical activity of a brain region and its capacity to process information (Li et al., 2022) is still missing although electrical activity of neurons has been investigated in experiments on single cells and their networks (Wu et al., 2019). The HH model represents the neuron as an electric circuit with a constant membrane capacitance in parallel with conducting ion channels (Nir et al., 2007). It became a gold standard for neuronal electrophysiology and is the basis of excitability through ionic currents, accurately representing the shape of spiking, its duration, activation thresholds, impedance, hyperpolarization, and the refractory period (Appali et al., 2012). The HH model respects Kirchhoff’s laws for electrical circuits and conducting cable theory. The membrane conductance is an empirical function of voltage and time while the resting membrane potential depends on the concentration gradients of ionic species, hence the nonlinear Goldman-Hodgkin-Katz equation, and not the Nernst equation, is more appropriate for its evaluation (Chrysafides et al., 2022). Membrane permittivity depends on its phospholipid composition and morphology, which may vary between neurons. The HH model still represents the most generally accepted conductance-based model in neurophysiology.

Electrophysiology measurements provided evidence for the neuronal membrane’s nonelectrical effects (thermodynamic and mechanical) of APs showing that during the depolarization phase heat is dissipated and then entirely reabsorbed during the re-polarization phase (De Lichtervelde et al., 2020). This is physically plausible assuming that an AP wave interacts with the membrane’s lipid bilayer adiabatically. During the AP, the neuronal membrane simultaneously increases its thickness and shortens longitudinally, resembling a mechanical pulse giving rise to a nerve pulse soliton propagation theory, which rests on thermodynamic and mechanical properties of membrane phospholipids (Wu et al., 2019). Fluctuations due to the lateral compression of the lipid chains cause pronounced changes in their elastic compressibility, bending stiffness, and relaxation times, leading to a nonlinear increase in compressibility, which is frequency dependent. This coupling together with nonlinearity, provides conditions suitable for soliton generation (Singh et al., 2021).

The brain’s approximately 100 billion neurons create a dynamic information field originating in electric currents and EMFs (Mac Iver, 2022). Their electrical activity generates three interconnected potential fields: the local field potentials (LFPs), the vector electric field, and the vector magnetic field (Hales et al., 2014). EMF polarizes the distribution of ionic charges, which then become ordered, causing changes in the membrane conductance modulating the transmembrane potential (Li et al., 2022). These information fields can act locally or lead to yet uninvestigated physical processes on a larger scale. The synchronous action potentials of about 50,000 pyramidal neurons generate an EMF that can be detected using magnetoencephalography (Mandal et al., 2018).

## Electromagnetic activity of the brain

Polarization and the magnetic induction effects of external EMF on the brain can have therapeutic effects. Repetitive transcranial magnetic stimulation has become a non-invasive treatment for a few psychiatric and neurological disorders, including major depression, epilepsy, Parkinson’s disease, and stroke rehabilitation (Peterchev et al., 2012). Transcranial electric stimulation delivers weak electrical currents through the scalp, and because of its safety, it is also used on healthy subjects to improve their declarative and working memory, verbal fluency, and planning ability (Chail et al., 2018). The molecular mechanisms of these effects involve both EMF interactions with individual neurons and the whole brain, and are related to an increased influx of Ca^++^ through the neuronal membrane (Sun et al., 2016). The magnetic field generates a force on moving ions exposed to EMF. This EM force also acts on the voltage-gated ion channels (VGCs), which are particularly susceptible to charge changes (Bertagna et al., 2021). Both acute and chronic exposure to EMF increases the rate of transport of Na^+^, Ca^++^, and calcium-activated potassium ions through VGCs, reorienting the phospholipid bilayer (Rosen, 2003). The permanent phospholipid dipoles are very sensitive to thermal excitation and EMFs; the former randomizes dipoles, while the latter align them (Lisi et al., 2006). The development of sensors composed of quantum defects within a diamond chip enabled precise measurements of the magnetic field correlated to an individual neuron’s AP (Petrini et al., 2022). Based on the thermodynamic properties of neuronal membranes, including volume and area changes and ion dynamics, APs can be described as a mechanically-coupled EM events rather than propagating voltage gradients (Wu et al., 2019). A significant challenge is to quantify the molecular mechanisms by which EMFs and mechanical forces are transformed into intracellular biochemical signals involved in yet unknown mechanisms responsible for the brain’s cognitive functions. Here we ask the following questions: What is the consequence of the change in the phospholipid composition on the intrinsic electrical properties of the neuronal membrane and neuronal function? Could this lead to a more integrated EM system with rich and tunable properties (Figure 1).

## Model assumptions and testable hypothesis

Membrane’s phospholipid bilayer composition and spatial distribution of its components allow for intracellular and extracellular fluid separation and selective transport across its thickness. It mainly contains phospholipids (phosphatidylcholine, phosphatidylethanolamine, phosphatidylserine, phosphatidylinositol, and sphingomyelin), cholesterol, gangliosides and cerebroside (Svennerholm et al., 1994). Between its two layers, phospholipids are asymmetrically distributed, and so are their head groups. Phosphatidylserine and phosphatidylinositol are negatively charged and are found preferentially at the inner leaflet contributing to the negative charge of the cytosol. Head groups are either zwitterionic, polar, or charged, therefore, being hydrophilic they are exposed to the aqueous medium. The electrical profile of excitable membrane lipids consists of a transmembrane potential and a boundary potential, the former determined by the ionic concentration gradient. The latter consists of a surface potential at the water-lipid interface and a dipole potential between the aqueous phase and the membrane’s aliphatic chain interior (Peterson et al., 2002). The phospholipid carbonyl group orientation and especially the lipid-bound water molecules determine the dipole potential (Clarke, 1997). We represent phospholipid head groups as moving dipoles interacting with their neighbors, whose synchronized oscillations can be excited by the AP’s electric field. Dipolar oscillations generate EMFs, which can lead to signals propagating both in the inter-neuronal milieu and into the cellular interior with downstream effects such as EM pattern formation and information storage.

**Figure 2:**
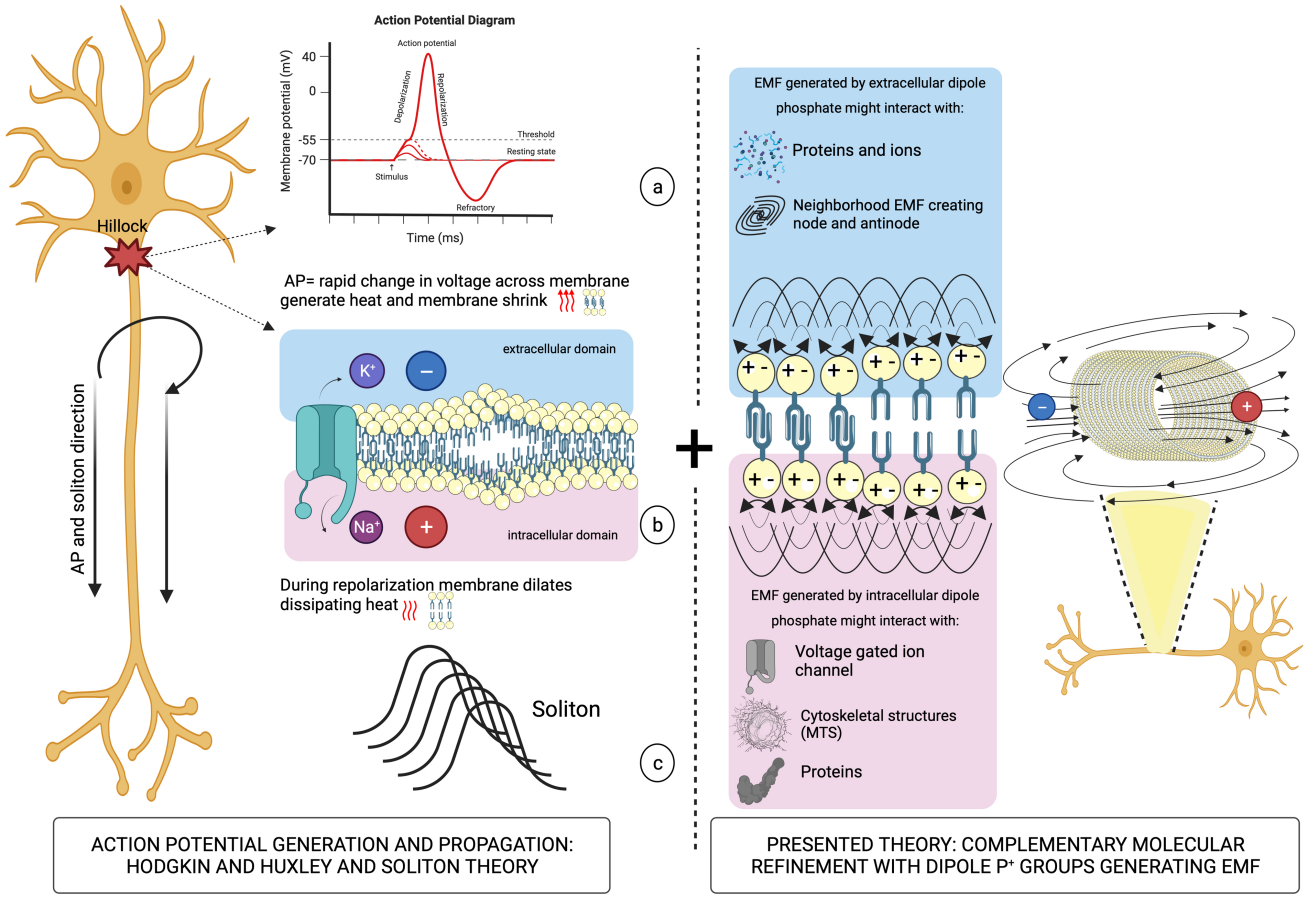
Left panel: schematic illustration of the AP propagation (a) causing mechanical deformation of the neuronal membrane (b) leading to soliton formation (c). Right panel: oscillations of P^+^ group dipoles that generate EMFs.

The molecular structure of the hydrophilic head groups of charged phospholipids involves phosphate (P^+^) bonds, whose orientation in the horizontal plane is an important degree of freedom (Simeonova and Gimsa, 2006). The partitioning of the P^+^ groups in the region between the aqueous phase and the aliphatic chain’s interior of the membrane allows for sensing of the local ionic potential, which interacts with the P^+^ causing rotational motion of phosphate head groups that generates dipole oscillations. EMFs acting locally on the asymmetrically charged membranes may finely modulate their electrical conductance by re-ordering ions and regulating the conformational states of protein channels (Peterson et al., 2002). Ionic currents flowing in the vicinity of P^+^ dipoles on the extracellular side of an axon could generate EMF coupled to the AP by changing the conformation of a voltage-gated Na^+^ channel. At the inner layer, EMFs could transfer information inside the cell by interacting with cytoskeletal structures, such as parallel bundles of microtubules (MTs) structurally stabilized by microtubule-associated proteins (Figure 2).

It is well-known that many neurodegenerative diseases are associated with defective MT cytoskeleton (Tuszynski, 2009), hence such defects could impair information transfer and storage in MTs.

**Figure 3:**
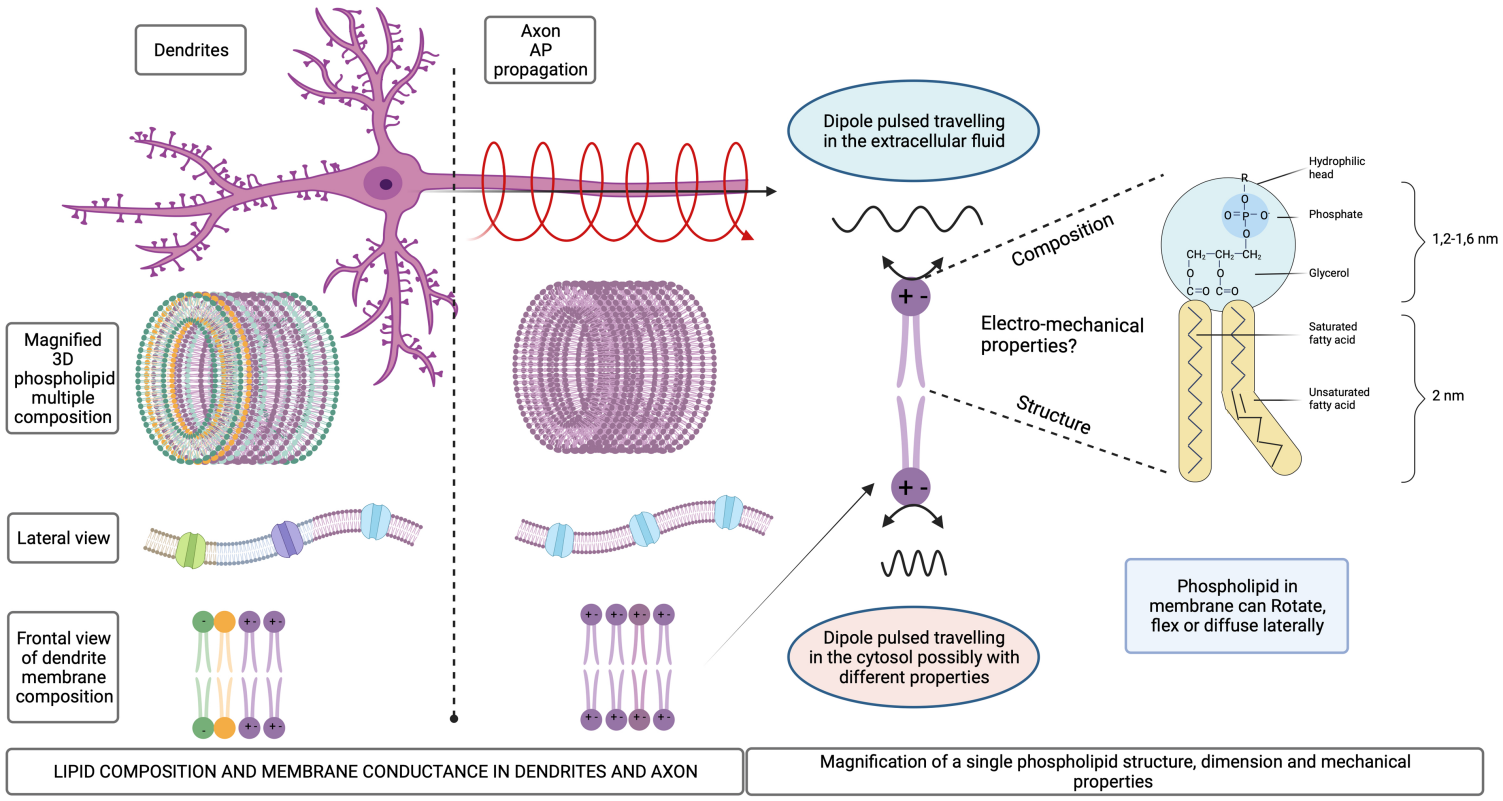
Schematic representation of different lipid compositions for dendrites and axonal membrane. Mechanical degrees of freedom of the phospholipid molecules in a membrane.

Quantitative characterization of the cellular membrane composition is still technically challenging due to the limited spatial resolution with an area of one micron squared containing approximately 100,000 phospholipids (Alberts et al., 2008). Their fatty-acid tails freely rotate around their axes, flex, and diffuse laterally within the single membrane leaflet. This mobility confers fluidity to cellular membranes, which can exhibit a phase transition involving the ordering of phospholipid tail groups influenced by temperature, pressure, pH, or voltage. As a result, lipid configurations change dramatically, and different membrane domains surrounding proteins contain either ordered or disordered lipids. Furthermore, the types of phospholipids found in membranes are very diverse according to the degree of aliphatic chain saturation, spacing and length. Also, the functional groups bonded to the P^+^ are very different electrostatically, most of them are negative. However, phosphatidylcholine and phosphatidylethanolamine head groups contain positive and negative charges being zwitterionic (Figure 3). Phosphatidic acid is negatively charged at physiological pH (Bohinc et al., 2022).

How structural composition of cellular membranes relates to function is inferred from individual phospholipid properties (Bagheri et al., 2020). The lipidomic studies proceed apace with the technical advances in spectroscopy and gas chromatography offering hope for detailed insights (Makowski, 2008). In the absence of definitive data, we assume that different lipid compositions in dendrites, soma, axon, and axon terminals are responsible for their different conductivities (Almog and Korngreen, 2014). Various physical mechanisms have been identified that involve the use of EMFs interacting with biological structures, which may result in the ultra-efficient storage, processing, and retrieval of information linked to specific biological functions (Rouleau and Dotta, 2014) such as neurotransmitter release, ion channel effects, memory encoding using CaMKII enzyme phosphorylation, and interactions of EMFs with MTs in neurons (Beck and Eccles, 1998). However, an integrated model is still needed to explain how EMF interactions are created.

Technology for the holographic image recording in photographic materials such as silver halide and photopolymer films has been known for decades (Kurtz et al., 1975; Lawrence et al., 2001). These recordings of interference fringes in a hologram bear no resemblance to the recorded original, yet they can reconstruct the encoded data, reproducing the original in 3D detail (Optical Holography: Principles, Techniques and Applications – P. Hariharan, P… Hariharan-Google Books, 2023). Holography provides an extremely efficient method of data storage, processing, and retrieval (Heanue et al., 1994). One of the most interesting properties of holograms is fractality, whereby each fragment of a hologram contains the entire information about the encoded image. Holography has been proposed as an explanation for the brain’s information storage capabilities earlier (Dolgoff, 1971). Using a mixed-system approach that combines classical and quantum theories pioneering work investigates the stability and non-local properties of memory, laying the foundation for the concept of quantum brain dynamics (Stuart et al., 1979).

Indeed, it is the best-positioned mechanism for the brain to accomplish what it does within limited space and metabolic energy usage (Petrini et al., 2022).

We formulate a holographic model based on the generation of coherent oscillations of neuronal membrane dipoles using the Froehlich mechanism of biological coherence, whose recent experimental evidence in protein systems makes it plausible (Lundholm et al., 2015; Nardecchia et al., 2018). While the Froehlich model is based on quantum interactions leading to Bose condensation of dipolar oscillations, this does not imply a strictly quantum mechanical process since long-range coherence can also be achieved using classical Maxwellian dynamics of interacting dipolar oscillations leaving the question of quantum versus classical foundations of the mechanism for future investigations.

We propose that membrane conductance variability for different neuronal parts and for different neuron types, is dependent on the lipid composition and spatial distribution. Hence, investigating the molecular composition of neuronal pyramidal cells in the neocortex could add invaluable insights into the biophysical mechanism of connecting electrical signals with perceptual binding. Pyramidal neurons in the neocortex have columnar distribution with a dendritic extent spanning multiple layers, integrating local cortical column computation. The generated signals travel through long axons transferring local information to distant cortex regions. Characterization of the pyramidal cell’s lipid composition is crucial as it may provide the mechanism by which cortical computational activity creates conscious experience.

## The electromagnetic model of the brain

Fröhlich’s theory of biological coherence was based on strong interactions between polar head groups of cell membranes. This is of major significance since dipole moments of neuronal membranes and the AP effects on them satisfy the assumptions required for the coherent state formation, namely:

- A continuous supply of metabolic energy above a minimum threshold required to achieve synchronization of membrane dipole oscillations.
- The presence of thermal noise due to physiological temperature.
- Internal structural organization.
- The existence of a large trans-membrane potential difference.
- Nonlinear interactions between oscillating dipoles.

These assumptions are satisfied by the neuronal membrane phosphate groups’ dipoles provided both AP energy and the rate of AP generation is sufficiently high. This externally supplied energy by APs is channeled into a single collective dynamical mode. Associated with this dynamical order is the emergence of electric polarization due to the biomolecular dipole interactions. The predicted coherent modes of dipole oscillations are in the 10^11^–10^12^ Hz. frequency range. The emergence of coherence among the phospholipid head groups is due to the membrane’s strong electric potential gradients on the order of 100 mV across the thickness of 5 nm giving an electric field intensity up to 20 × 10^6^ V/m. The resultant dipole–dipole interactions are predicted to propagate with a velocity of about 10^3^ m/s, much faster than AP propagation. Important individual differences due to the lipid composition of the membrane will substantially affect the properties of the coherent dipolar waves. Maxwell’s theory of electromagnetism shows that oscillating dipoles emit EMFs. Therefore, neuronal membranes, with their coherently oscillating dipoles due to (incoherent) energy input from APs, should produce EMFs in the process.

Reimers et al. (2009) re-analyzed the conditions for the Bose condensations of biological dipole oscillations and classified Fröhlich condensates into 3 types: (a) weak, (b) strong, which an extremely large amount of energy is channeled into one vibrational mode, and (c) coherent whose energy is funneled into a single quantum state. Only weak condensates can be produced from biochemical energy inputs, and they require the pumping rate for biochemical energy above the threshold of (100 K) k_B_/s where K is degree kelvin and k_B_ is the Boltzmann constant. The rate of energy loss due to thermal interactions must be less than 20 K k_B_/s, i.e., 5 times lower than the rate of energy supply. In this case, the condensate can form on a 10–1,000 ns time scale. At the physiological temperature of 310 K, this condition translates into energy supply exceeding (1/3) k_B_T/s. Below we show that APs provide such conditions for neuronal membrane dipoles. We first estimate the interaction energy between two neighboring dipoles in a phospholipid bilayer. The dipole moment of each phosphate head group is approximately 20 debye, so the dipole–dipole interaction, assuming a 5-angstrom distance between the centers of these dipoles is on the order of 9 k_B_T. Each group has several neighbors, so the total dipole interaction energy exceeds thermal energy by a significant factor. Next, we calculate the strength of the interaction energy between the AP and the phospholipid head group dipoles. APs travel at speeds between 0.5 and 100 m/s and are generated at a rate between 10 and 100 per second, so the average time between the arrival of APs at a given point in the neuronal membrane varies between 10 and 100 ms. The duration of each event is approximately 2 ms over which time the voltage across the membrane, V, varies between – 70 mV and + 40 mV. We approximate the potential difference by 100 mV, which is 4 times larger than thermal energy k_B_T, experienced by a single electric charge, since k_B_T = 25 meV. Then, an estimate of the interaction energy between the AP and a phospholipid dipole moment is: E_ap-d_ = − (dV/dx). Since dx is the membrane thickness, about 4 nm, we find that E_ap-d_ = 0.5 k_B_T. We then calculate the rate of energy pumping, S, knowing the rate of arrival of APs as:


$$5\;k_{B}T/s<S=E_{ap-d}/dt<50\;k_{B}T/s$$

This value clearly exceeds the minimum requirement of 0.3 k_B_T/s, hence we conclude that a weak Bose-Einstein condensate can be generated in the dipolar system of neuronal membrane’s head groups. It is worth noting that Raman scattering studies (Leonov et al., 2019) demonstrated the existence of two absorption peaks in phospholipid head groups in the visible and near infrared range, namely: 1.25 mm wavelength (1.0 eV) and 590 nm wavelength (2.1 eV) and measured the acoustic wave propagation through the membrane at a speed of 2.4 km/s. These results provide indirect support to the concept of EMF propagation in ordered membrane dipoles with an order of magnitude estimate of the characteristic EMF wavelengths as well as the propagation velocities of the electro-mechanical excitations in neuronal membranes.

## Holographic image formation

**Figure 4:**
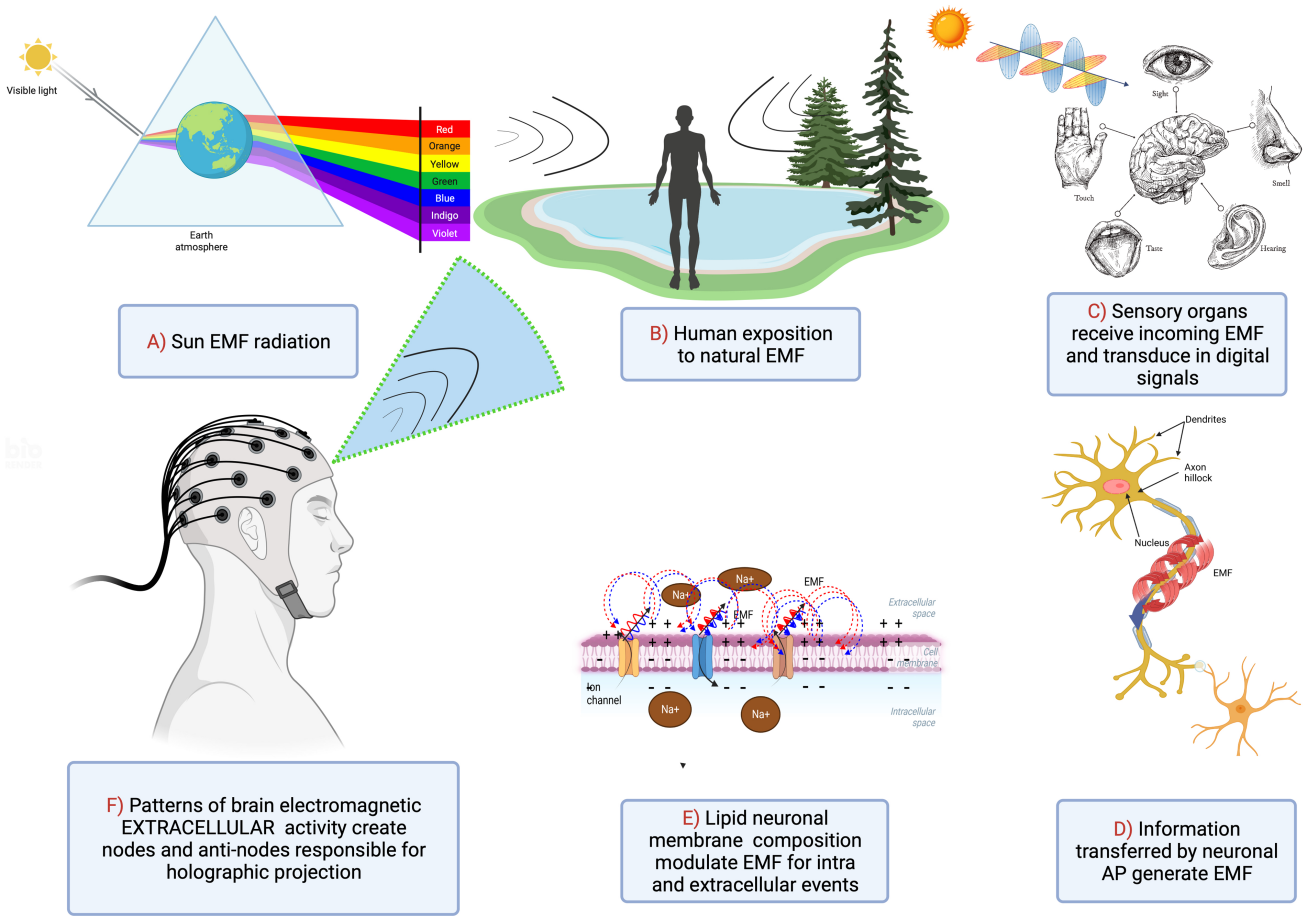
Integration of sensory inputs into the synchronized image of the outside world via neuronal activation and subsequent generation of EMF patterns.

Formation of holographic images is achieved by constructive and destructive interference patterns of two propagating EMFs. We hypothesize that a coherent EMF produced by dipole oscillations in the membrane of one neuron can interfere with a similar EMF produced by a neighboring neuron. One of these waves can be considered a reference beam while the other an object beam using holographic terminology. This will create a pattern of nodes and antinodes due to EMF interference. Importantly, within the neocortex, multiple pyramidal cell axons are almost parallel, capable of producing multiple cylindrical waves that overlap and interfere with each other. Consequently, instead of a fringe pattern of equally spaced parallel lines, the resulting interference pattern will appear much like a conventional hologram projecting a 3D image on a screen. A magnified hologram’s interference pattern, resulting from multiple complex beams interfering with each other could be formed when a number of neurons synchronously fire producing coherent dipole waves that in turn create multiple interfering EMFs (Figure 4).

As the first step toward model validation, we propose to combine *in-silico* modeling and design of quantum optics experiments aimed at detecting these EMF effects. Atomic-level models of membranes can be used to investigate the propagation of solitons and dipolar oscillation patterns. Extremely sensitive quantum optics techniques are available for detecting single EMF quanta. As an additional verification of the hypothesis, it is important to investigate the role of anesthetic molecules since anesthesia is known to reversibly abolish consciousness. With the presence of anesthetic molecules, we expect the membrane to change its mechanical and electrical properties affecting AP propagation and leading to the elimination of holographic images. Changes of the mechanical properties of membranes due to anesthetic binding have been recently computed at an atomistic scale. With the membrane’s bending modules being on the order of 45 k_B_T, anesthetic molecules reduce it by as much as 40% (Zizzi et al., 2022). Calculations of the electromechanical waves propagating through the membrane with/without anesthetic molecules can establish if this mechanism of anesthetic action is capable of effects such as abrogating the dipolar oscillations of neuronal membranes or slowing down or even stopping electro-mechanical soliton propagation.

## Conclusion

EMFs have been found to be generated within the brain, but more research needs to be done to understand how the brain uses these EMFs. We hypothesize that due to the stimulation of membrane dipoles by APs, which carry electric field gradients, coherent dipole oscillations are formed under suitable conditions. These oscillations satisfy the weak Bose-Einstein condensate criteria of the Froehlich model of biological coherence and take place among the polar head groups of phospholipids. They can subsequently generate EMFs. Neighboring neurons that fire synchronously can then create interference patterns offering the plausibility of 3D holographic image formation. Such holographic images were proposed in general terms in Holonomic Brain Theory without providing a mechanistic model (Pribram and Carlton, 1986).

Nishiyama incorporates the Lagrangian density of the ϕ4 model and extends to quantum field theory (QFT) in multiple layers representing the cortex of the brain, with potential applications in manipulating quantum fields and holograms in the brain (Nishiyama et al., 2023).

Our model is a concrete mechanistic formulation of this theory. If experimentally validated, this could elucidate numerous empirical observations in the area of neurology and psychopathology. For example, membranes with structural damage or unbalanced phospholipid composition could generate distorted images which result in perceptional distortions of reality in schizophrenia. Neurodegenerative diseases such as Alzheimer’s could be directly related to defective memory storage in neuronal microtubules in the case of their destabilization by MAP tau hyperphosphorylation. Anesthesia affects membrane receptors but also causes structural changes in the membrane such as loss of mechanical rigidity and changes in thickness and spatial distribution of phospholipids. These effects could be directly linked to the loss of coherence of the interacting dipole oscillations in the membrane. Moreover, encoding information in microtubules by phosphorylation of their C-termini as proposed elsewhere (Craddock et al., 2012) could provide a microscopic explanation of memory formation using EMFs directly affecting neuronal cytoskeleton whose electrical properties have recently revealed remarkable wealth of behavior (Santelices et al., 2017; Kalra et al., 2020). For example, MTs possess transistor-like (Priel et al., 2006) and memristor-like properties (Tuszynski and Douglas, 2020). They could be involved in modifying the interactions between the membrane, ion channels and APs (Singh et al., 2021). Finally, our model is consistent with the Integrated Information Theory (IIT; Tononi, 2017) of consciousness since the mechanism we propose involves sensory inputs carrying information about the external reality, which then produce synchronized images in a virtual reality generator at the level of neuronal assemblies. Furthermore, our model is consistent McFadden’s electromagnetic information field theory, which claims that thoughts are electromagnetic representations of neuronal information in the brain (McFadden, 2020).

## Data availability statement

The original contributions presented in the study are included in the article/supplementary material, further inquiries can be directed to the corresponding author.

## Author contributions

MC: Conceptualization, Writing – original draft, Writing – review & editing. MD: Writing – original draft, Writing – review & editing. JT: Supervision, Writing – original draft, Writing – review & editing.
